# Sustainable Model for Public Health Emergency Operations Centers for Global Settings

**DOI:** 10.3201/eid2313.170435

**Published:** 2017-12

**Authors:** S. Arunmozhi Balajee, Omer G. Pasi, Alain Georges M. Etoundi, Peter Rzeszotarski, Trang T. Do, Ian Hennessee, Sharifa Merali, Karen A. Alroy, Tran Dac Phu, Anthony W. Mounts

**Affiliations:** Centers for Disease Control and Prevention, Atlanta, Georgia, USA (S.A. Balajee, O.G. Pasi, P. Rzeszotarski, T.T. Do, I. Hennessee, K.A. Alroy, A.W. Mounts);; Cameroon Ministry of Health, Yaoundé, Cameroon (A.G.M. Etoundi);; Synergy America, Inc., Duluth, Georgia, USA (S. Merali);; Vietnam Ministry of Health, Hanoi, Vietnam (T.D. Phu)

**Keywords:** Disease management, disease outbreaks, emergencies, epidemics, epidemiology, global health, emergency operations center, policy, public health, surveillance, Cameroon, United States, Vietnam

## Abstract

Capacity to receive, verify, analyze, assess, and investigate public health events is essential for epidemic intelligence. Public health Emergency Operations Centers (PHEOCs) can be epidemic intelligence hubs by 1) having the capacity to receive, analyze, and visualize multiple data streams, including surveillance and 2) maintaining a trained workforce that can analyze and interpret data from real-time emerging events. Such PHEOCs could be physically located within a ministry of health epidemiology, surveillance, or equivalent department rather than exist as a stand-alone space and serve as operational hubs during nonoutbreak times but in emergencies can scale up according to the traditional Incident Command System structure.

Every country needs a system for responding to emergencies and managing emergency response. Emergency Operation Centers (EOCs) are increasingly viewed as necessary components of emergency preparedness and are used for multiagency coordination and response to a variety of hazards, including natural disasters, chemical spills, radionuclear incidents, humanitarian emergencies, and disease outbreaks. Public health EOCs (PHEOCs) are physical spaces with the ability to monitor events using various sources of data, improve communication between public health and emergency management personnel, facilitate coordination with multiple response partners, and provide space for members of the incident command team to gather and work (*1*–*7*).

When activated, a PHEOC is a location for the coordination of information and resources and is staffed with teams of subject matter experts, analysts, logisticians, and support staff (*2*,*3*). During activation, PHEOCs monitor epidemiologic data and field reports from a variety of sources using data technologies and informal networks of public health professionals (*1*,*8*). Scalability is essential for maintaining the effectiveness of a PHEOC (*2*), and it can be partially or fully activated according to situational needs (*9*). When inactive, many PHEOCs reduce in size or become dormant, and routine surveillance activities continue elsewhere within a ministry or department of public health (*3*,*6*,*10*).

In the United States, the Centers for Disease Control and Prevention (CDC) has a 24,000-square-foot PHEOC staffed by trained personnel 24 hours per day, 365 days per year, on CDC’s main campus in Atlanta, Georgia (*1*). The CDC PHEOC may be notified about potential public health threats through its watch desk, which receives calls primarily from clinicians and other state and local entities, including PHEOCs. Notification also can come from public health partner briefings, field operations intelligence, reports from media and the Internet, and the International Health Regulations reporting system maintained by the World Health Organization (WHO) (*11*).

Although the CDC PHEOC houses a unit that monitors a wide variety of media sources for reports of outbreaks, most routine domestic surveillance data are collected and analyzed by the states and individual pathogen- or disease-specific programs within CDC. For instance, CDC’s Influenza Division collects, compiles, and analyzes information about influenza activity year-round in the United States. This information is communicated to the public in FluView (*12*), a weekly influenza surveillance report, and FluView Interactive (*13*), which enables in-depth exploration of influenza surveillance data. The CDC PHEOC can access and view FluView 24/7 but relies on experts in the Influenza Division to analyze and interpret data and identify major aberrations. If an aberration in the data was thought to represent an event with public health consequences, such as the emergence of a new influenza virus rapidly spreading among humans, the PHEOC would be activated and all influenza surveillance activities moved into it during the period of activation.

Since its official launch in 2003, the CDC PHEOC has been central to CDC’s timely and efficient coordination of public health threats and has responded to ≈60 domestic and international public health threats, including hurricanes; foodborne disease outbreaks; the 2009 A(H1N1) influenza pandemic; the Haiti cholera outbreak; and the outbreaks of Middle East respiratory syndrome, Ebola virus infection, and Zika virus infection (*9*). Although the CDC PHEOC has been a successful model in the United States, it might be less relevant for resource-limited countries. Maintaining a freestanding, constantly staffed PHEOC with a large dedicated workforce might be prohibitively expensive. In addition, recruiting a highly skilled epidemiologic workforce for an EOC might be challenging in these countries. Furthermore, the CDC PHEOC conducts surveillance on a global scale, whereas some countries may prioritize a more regional or national focus and thus might not have the ability or the need to scale up human and technical resources to tackle public health threats on the international stage.

Countries face challenges with surveillance and outbreak response because of 1) fragmented data streams that do not enable easy access to raw data for timely analyses and data use, 2) a small workforce that is responsible for most surveillance and response-related activities, 3) poor coordination during outbreaks resulting in slow response, and 4) limited resources dedicated to public health (*4*,*10*,*14*,*15*). To mitigate these challenges, PHEOCs in global settings can serve as epidemic intelligence hubs by receiving, analyzing, and visualizing multiple data streams, including surveillance data, and being staffed with a trained workforce capable of analyzing and interpreting data in real time. Such PHEOCs can be embedded within a ministry of health epidemiology, surveillance, or equivalent department, rather than existing as a standalone space, and can operate continuously for routine health surveillance.

The Global Health Security Agenda (GHSA), officially launched in 2014, was developed to strengthen countries’ capacity to prevent, detect, and respond to human and animal biologic threats (*16*,*17*). The 5-year target for GHSA’s Emergency Operations Centers Action Package is that “Every country will have a public health Emergency Operations Center functioning according to minimum common standards; maintaining trained, functioning, multi-sectoral rapid response teams (RRTs), ‘real-time’ bio-surveillance laboratory networks and information systems; and trained EOC staff capable of activating a coordinated emergency response within 120 minutes of the identification of a public health emergency” (*18*).

With the launch of GHSA and the need to develop PHEOCs and surveillance response capacities in countries around the world, we outline a sustainable model for PHEOC operations. Such PHEOCs will operate continuously by maintaining routine surveillance activities and serving public health needs during outbreak and nonoutbreak periods, thereby ensuring sustainability and helping address other national needs, such as routine analyses and use of surveillance data. We illustrate this approach with 2 case studies.

## Case Study 1: Vietnam

Vietnam has 4 technically strong regional institutes with moderately advanced laboratory and epidemiologic capacity, resulting in scores of 3 or 4 out of 5 for laboratory and surveillance capacity indicators using the Joint External Evaluation of the International Health Regulations Core Capacities tool (*19*). These institutes oversee public health activities in their respective regions (North, South, Central Coast, and Central Highland), including the response and management of outbreaks that are beyond the capacity of local health departments. Nationally, the General Department of Preventive Medicine (GDPM), an agency within the Ministry of Health, provides public health policy and the strategic direction of public health activities, including surveillance. The GDPM developed a national PHEOC with the support of CDC and the US Defense Threat Reduction Agency’s Cooperative Biologic Engagement Program as part of a GHSA demonstration project in 2013. Since then, the PHEOC has been used to manage responses and risk assessments to several different threats, including a nationwide measles outbreak, concerns about the importation of Ebola virus infection and Middle East respiratory syndrome, and recently, the emergence of Zika virus infection. The national PHEOC conducted and coordinated several training sessions for Ministry of Health and regional institute personnel on basic Public Health Emergency Management, facilitated participation for GDPM and regional institute staff in CDC’s Public Health Emergency Management Fellowship training program, and has conducted several tabletop exercises and drills. A comprehensive PHEOC operational handbook was also developed and recently disseminated throughout the country’s public health system (*20*).

Vietnam has several surveillance systems that generate data from a variety of sources. Hospitals are required to routinely report notifiable diseases, including several high-risk illnesses that must be reported within 24 hours. Typically these data are transmitted through the public health system from communes and districts to the province level, and then the regional institutes, through aggregated reports, submit these data to an electronic Communicable Disease Surveillance software. Since July 1, 2016, the Ministry of Health has been rolling out a system of case-based reporting on the established backbone of aggregated data reporting. In addition, multiple separate sentinel surveillance networks monitor for Japanese encephalitis virus; hand, foot, and mouth disease; influenza-like illness; severe acute respiratory infections, and dengue virus infection. Each system has an independent reporting mechanism, but all are monitored by the same small group of regional institute–level epidemiologists. Surveillance for malaria, tuberculosis, and HIV infection also have separate reporting systems. Each regional institute has a public health laboratory system, but the laboratories are not directly connected to the epidemiology or disease control departments that monitor for outbreaks. In addition to these indicator-based surveillance systems, event-based surveillance systems recently have been improved in Vietnam, where community health workers and healthcare providers can report unusual events through public health reporting networks. The fragmentary nature of the surveillance data available through diverse reporting sources impedes timely detection of outbreaks, making the creation of integrated data systems critical to the success of these PHEOCs.

To help mitigate these challenges, the Vietnam Ministry of Health envisioned a network of PHEOCs that will be an interlinked system of information hubs, one at each regional institute. Each PHEOC will be connected to the network through its own data warehouse, which is in turn connected to the national data warehouse at the national PHEOC at GDPM. The warehouses incorporate and integrate data from multiple surveillance sources and enable analyses with the District Health Information System 2 software platform (*21*). For immediate accessibility, data dashboards with automated analyses are being created for each high-priority disease, enabling surveillance staff to instantly see the status of disease cases in their region. Alert thresholds for specific endemic seasonal diseases, such as dengue and influenza, have been designed to trigger notifications to the regional institutes.

The National Institute of Hygiene and Epidemiology (NIHE) in Hanoi and the Pasteur Institute of Ho Chi Minh City (PI-HCMC) lead the surveillance and outbreak response for the North and South regions, respectively, and collaborate with GDPM. NIHE has completed the establishment of a PHEOC, and PI-HCMC is in the process of doing the same. Vietnam plans to develop 2 additional PHEOCs in the remaining regional institutes in 2017. Both PHEOCs (NIHE and PI-HCMC) are situated physically and administratively in departments of epidemiology or disease control at the regional institutes and are staffed by epidemiologists within those departments, the same epidemiologists responsible for routine surveillance. A small number of support staff, including full-time PHEOC managers and information technology staff, are being recruited. During nonoutbreak times, the PHEOCs will be surveillance hubs where data from notifiable disease reporting from healthcare facilities, sentinel surveillance sites, and public health laboratory systems are all available through the data warehouse and displayed on data dashboards that automate routine analyses. Epidemiologists in each PHEOC will monitor and interpret the various streams of surveillance data to define usual patterns of disease transmission and monitor for aberrations. Data also are summarized for weekly distribution to policy makers in the MOH. The PHEOCs also will receive and incorporate event reports from the media, community, healthcare facilities, and event-based surveillance systems enabling more timely detection of emerging or small outbreaks. Separate real-time data dashboards are in place for priority diseases, such as Zika virus infection (Technical Appendix Figure).

After WHO declared Zika virus infection as a Public Health Emergency of International Concern, the national PHEOC at GDPM began operating as a nerve center for Zika virus preparedness and response (*22*). Through the institution of a national preparedness and response plan, ongoing data surveillance, and multiagency meetings, the Vietnam PHEOC network has monitored and documented the Zika epidemic in the Americas and tracked cases within Vietnam. The Vietnam PHEOCs also are training centers for Vietnam’s Field Epidemiology Training Program (FETP). That program recently inducted full-time fellows for the first time in 2016. These fellows rotate through the PHEOCs, where they are responsible for analyzing surveillance data and writing data summaries.

Ultimately, the development of Vietnam’s PHEOC policies and operating procedures had to be tailored to the specific context of the country’s existing legislative background. Formal PHEOC activation at CDC mobilizes financial resources for outbreak response and mobilizes personnel from other departments within the organization, which expedite the processes usually required for travel authorization and the clearance of communications materials. In Vietnam, however, these same actions are accomplished by the formal declaration of an “outbreak,” which carries a specific legal meaning. This legislation, which long preceded development of a PHEOC, had to be taken into account when the EOC guidelines and manual of operations were drafted.

## Case Study 2: Cameroon

Cameroon has experienced nearly annual cholera outbreaks and 3 separate outbreaks of measles in 2014 and continues to encounter major challenges in containing these outbreaks. Obstacles to efficient containment of outbreaks include reporting lags from the field, delays in information sharing of outbreak data through the public health system, inefficient coordination of outbreaks, and slow response at the central level (*23**,**24*).

The Integrated Disease Surveillance and Response (IDSR) system is the framework for Cameroon’s disease surveillance and response. Public health policies, supervision and management of the health system, and IDSR at the central level are the responsibility of Ministère de la Santé Publique (MINSANTE), the Cameroon Ministry of Health. Cameroon has 10 regions with regional health delegations, and each is responsible for public health surveillance and response. Each region is further divided into districts, and the districts are additionally divided into health center catchment areas. These health center catchment areas are the outmost peripheral health units and may have community health volunteer networks. Aggregated reports of IDSR notifiable diseases are sent weekly from the districts to MINSANTE, and the process is completed by manual data entry shared by email.

In 2014, Cameroon began developing a PHEOC, and MINSANTE prioritized its establishment to improve outbreak coordination, management, and response. The PHEOC was developed in the capital city, Yaoundé. It was created after several trainings of MINSANTE personnel, including training on the Incident Management System, participation in CDC’s Public Health Emergency Management Fellowship training program, and the execution of several tabletop exercises and simulations. This knowledge was shared within the country, through a course taught by the newly hired PHEOC manager, with support from CDC subject matter experts.

The PHEOC was activated in May 2016 in response to an avian influenza virus A(H5N1) outbreak on a poultry farm in Yaoundé to enable the early detection of human cases, respond rapidly to interrupt human transmission, and oversee case management. A veterinary FETP fellow served as the liaison between the PHEOC and MINEPIA, Cameroon’s Ministry of Livestock, Fisheries, and Animal Industries, coordinating seamless communication between the National Veterinary Laboratory and the PHEOC. When the PHEOC was deactivated in June 2016, none of the human contacts had tested positive (*25*).

During the avian influenza outbreak, the PHEOC faced a challenge in securing Tamiflu (oseltamivir phosphate) (Genentech, South San Francisco, CA, USA), an antiviral medication used to treat persons with symptoms caused by influenza. Early in the PHEOC’s activation, all existing national stocks of Tamiflu were recognized to have expired, leaving the country unprepared for human cases. Working with WHO, the PHEOC obtained Tamiflu.

When GHSA was launched in Cameroon, MINSANTE understood that Cameroon could not wait for another outbreak and needed to begin operating the national PHEOC immediately. MINSANTE positioned the PHEOC as a hub to coordinate resources, information, and communication for data receipt, integration, analyses and interpretation, and coordination, with less focus on the physical infrastructure. Thus, the PHEOC runs out of a small multipurpose room within the MINSANTE facility, and a new facility is being built nearby. The lack of a dedicated physical place has not hindered the PHEOC’s operation. In 2016 alone, the PHEOC responded to a cholera outbreak; prepared for a Lassa fever outbreak when it broke out in neighboring Nigeria; responded to measles, monkeypox, and avian influenza virus A(H5N1) outbreaks; elaborated on contingency plans for Zika virus; fine-tuned monkeypox plans when human cases and fatalities were registered in neighboring Central African Republic; and preventively activated for wild poliovirus detected in Nigeria. Most recently, the PHEOC responded to a train derailment in the Ezeka district, demonstrating all-hazards response capability. All of these opportunities helped Cameroon improve its preparedness, reducing its response time from 8 weeks to 24 hours during the recent H5N1 influenza outbreak (Table).

**Table:** PHEOC activations illustrating improvements in time to activation, Cameroon, 2015–2016*

Date	Outbreak/disaster	Type	Event/outbreak location	Activation time	Comments/action taken
2015 Nov–2016 Jan	Cholera	Infectious disease outbreak	Gider District, Cameroon	8 wk	Major delays because of lack of coordination and accountability
2016 Mar–Apr	Measles	Infectious disease outbreak	Cameroon	4 wk	Delays because of lack of accountability
2016 May–Jun	Influenza A(H5N1)	Infectious disease outbreak	Cameroon	24 h	Benefit from lessons learned for first time. EOC’s Incident Manager is ministry of health staff member who graduated from CDC PHEOC fellowship
2016 Aug	Lassa fever	Infectious disease outbreak	Nigeria	2 wk	Outbreak in neighboring country provided opportunity to test preparedness and set up contingency plans for bordering districts
2016 Aug	Monkeypox	Infectious disease outbreak	Cameroon	24 h	Collaboration between CDC, Defense Threat Reduction Agency, and WHO to provide PPE to government of Cameroon
2016 Sep	Zika virus	Infectious disease outbreak	Latin America	2 wk	New opportunity to test preparedness and set up national contingency plan
2016 Oct	Camrail train accident	National disaster	Ezeka, Cameroon	1 h	Using PHEOC for other public health–related events that are not infectious diseases
2016 Oct	Monkeypox	Infectious disease outbreak	Central African Republic	24 h	Outbreak with human cases and deaths
2016 Nov	African Women Cup of Nations	National major event	Limbé and Yaoundé, Cameroon	Pre-event	Centre and Littoral Regions’ rapid response teams

*CDC, Centers for Disease Control and Prevention; EOC, Emergency Operations Center; PHEOC, public health EOC; PPE, personal protective equipment; WHO, World Health Organization.

Engaging Cameroon’s FETP within the newly created PHEOC was a critical component of the design of the country’s PHEOC. The FETP trainees are forming the critical workforce that regularly analyze IDSR data from the district, interpret results, and present the results to stakeholders each week. These epidemiologic meetings are led by FETP trainees at the PHEOC and include stakeholders from WHO, UNICEF, the National Public Health Laboratory, Centre Pasteur of Cameroon, International Medical Corps, Metabiota, MaSanté, CDC, various officers from concerned directorates, and surveillance teams from MINSANTE, among others. This ethos of cooperation and stakeholder engagement was crucial for coordination meetings later, during the H5N1 influenza activation.

As the concept of incident command started to take shape, it became apparent that 2 major gaps in the Cameroon health system had been secondarily bridged: more accountability and better coordination. The lack of these 2 attributes previously were the major cause of poor initial response to the wild poliovirus outbreak (2013–2015) (*26*).

Cameroon’s PHEOC faces many challenges, including a time lag in data availability from districts because of manual collection and reporting of data and limited information systems capacity to collect and analyze information from diverse data sources. To address this challenge, MINSANTE is investing in a data warehouse and an automated software platform at the district, regional, and national levels to make data available in near real time to decision makers at each level and to enable information flow into the PHEOC. Work is also under way to build capacity for automated data analysis and visualization at the PHEOC.

## A Sustainable, Optimal, and Continuous Use Model for PHEOCs for Global Settings

Developing PHEOCs to facilitate appropriate coordination, response, and management of public health events is essential for building countries’ emergency response capacity. Experience gained from developing PHEOC capacity in Vietnam and Cameroon demonstrated the following as a recommended sustainable path for PHEOC development:

1. PHEOCs benefit from being housed physically and administratively in close proximity to or within the epidemiology or surveillance departments of the ministry of health. This closeness establishes the PHEOC as a working hub readily accessible by epidemiologic staff.
2. PHEOCs should be epidemic intelligence hubs to receive, interpret, and visualize surveillance data from multiple sources. These hubs make information systems development a critical part of PHEOC operations. Mechanisms should be created that integrate data streams and develop data dashboards, automate routine analyses to improve the value and utility of surveillance data, and establish the continuous operations of the PHEOC.
3. Rotating FETP trainees through the PHEOCs provides the epidemiologic workforce needed for analysis and interpretation of surveillance data. This rotation can augment epidemiology workforce capacity, especially in ministries of health where epidemiology staffing is limited. It also provides a valuable training experience for FETP fellows.
4. PHEOCs should function during nonoutbreak periods, and surveillance data should routinely be interpreted by an epidemiologic workforce. Such an “always on” PHEOC facilitates the rapid transition to response mode during outbreaks and improves the cost-effectiveness of the infrastructure investment. Routine use of PHEOCs during outbreaks and during nonoutbreak periods helps ensure sustained technical capacity for data analyses, interpretation, and visualization tools and equipment, as well as the knowledge to analyze and interpret incoming health information.
5. Each PHEOC must be tailored to the legislative context in which it is situated. The result is a PHEOC that fits within local legislation and more fully meets the needs of the ministries of health.

The 2015 WHO Framework for a Public Health Emergency Operations Centre provides valuable information about the role, function, and construction of PHEOCs (*2**,**7*). A critical gap exists in guidance, however, regarding how PHEOCs maintain readiness between periods of activation. This gap in guidance is particularly relevant for resource-limited nations that might not be capable of readily scaling up human resources and technical capacity in the event of an emergency. It is more sustainable for PHEOCs in these countries to initially be established in departments or institutions that are already responsible for monitoring public health data and responding to disease outbreaks. Illustrations from Vietnam and Cameroon present the implementation of this approach and its associated successes and challenges.

The approach described here could enable rapid establishment of a PHEOC with minimal infrastructure and available workforce. Such a PHEOC will serve well in resource-limited settings as a continuously operational hub for surveillance, yet ready for activation during emergencies. As additional resources become available, this PHEOC model can expand to fit international standards and be capable of addressing all emergency hazards.

Technical Appendix Data dashboard in national public health emergency operation center for Zika virus, Vietnam.
