# Supplementary material for: Sustainable Model for Public Health Emergency Operations Centers for Global Settings
Source: Emerg Infect Dis. 2017 Dec;23(Suppl 1):S190–5. doi: 10.3201/eid2313.170435 (PMC5711308; doi:10.3201/eid2313.170435)
Supplement: Technical Appendix — Data dashboard in national public health emergency operation center for Zika virus, Vietnam. [file 17-0435-Techapp-s1.pdf]

Article DOI: https://doi.org/10.3201/eid2313.170435

Sustainable Model for Public Health Emergency Operations Centers for Global Settings

Technical Appendix

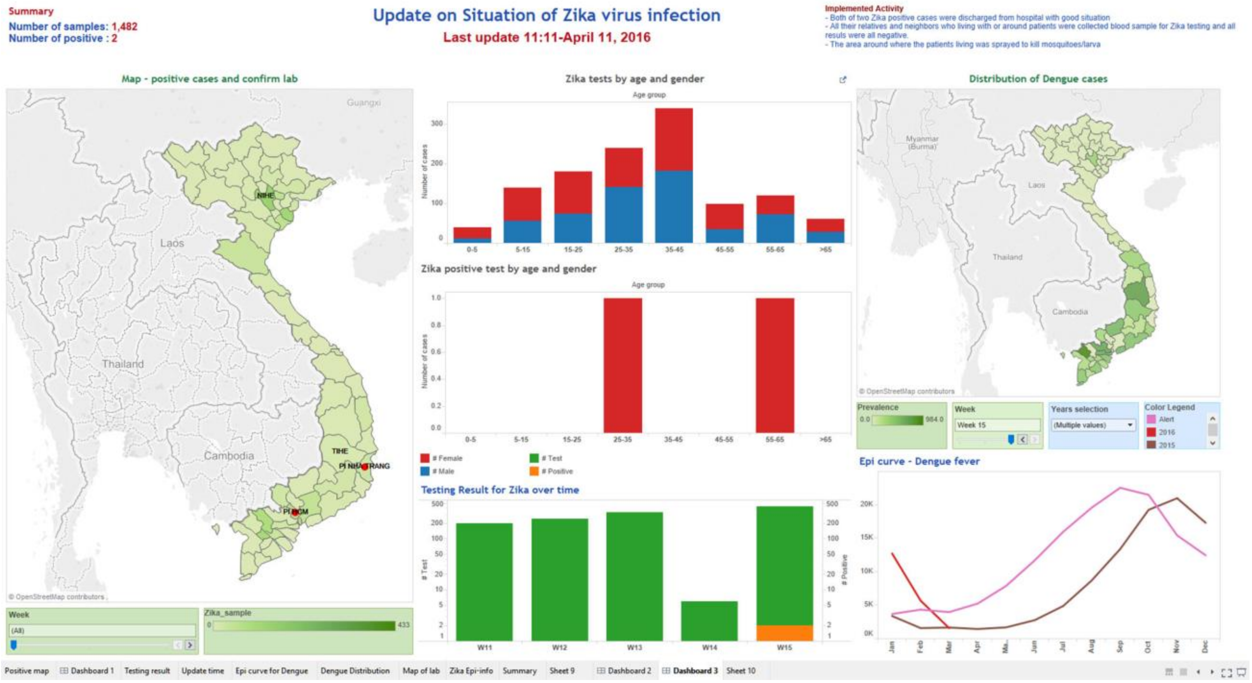

Technical Appendix Figure. Data dashboard in national public health emergency operation center for Zika virus, Vietnam.
